# Identifying Violent Behavior Using the Oxford Mental Illness and Violence Tool in a Psychiatric Ward of a German Prison Hospital

**DOI:** 10.3389/fpsyt.2019.00264

**Published:** 2019-04-23

**Authors:** Vincent Negatsch, Alexander Voulgaris, Peter Seidel, Robert Roehle, Annette Opitz-Welke

**Affiliations:** ^1^Charité–Universitätsmedizin Berlin, corporate member of Freie Universität Berlin, Humboldt-Universität zu Berlin, and Berlin Institute of Health, Institute of Forensic Psychiatry, Berlin, Germany; ^2^Universitätsklinikum Hamburg-Eppendorf, Institute of Sexual Medicine and Forensic Psychiatry, Hamburg, Germany; ^3^Department of Psychiatry and Psychotherapy, Prison Hospital Berlin, Berlin, Germany; ^4^Charité–Universitätsmedizin Berlin, corporate member of Freie Universität Berlin, Humboldt-Universität zu Berlin, and Berlin Institute of Health, Institute of Biometry and Clinical Epidemiology, Berlin, Germany; ^5^Charité–Universitätsmedizin Berlin, corporate member of Freie Universität Berlin, Humboldt-Universität zu Berlin, and Berlin Institute of Health, Coordinating Center for Clinical Studies, Berlin, Germany; ^6^Berlin Institute of Health (BIH), Berlin, Germany

**Keywords:** violence, prison, forensic psychiatry, schizophrenia, bipolar disorder, prediction tool

## Abstract

**Background:** Although there is evidence that individuals who suffer from severe mental disorders are at higher risk for aggressive behavior, only a minority eventually become violent. In 2017, Fazel et al. developed a risk calculator (Oxford Mental Illness and Violence tool, OxMIV) to identify the risk of violent crime in patients with mental disorders. For the first time, we tested the predictive validity of the OxMIV in the department of psychiatry at the prison hospital in Berlin, Germany, and presented findings from our internal validation.

**Materials and Methods:** We designed a cohort study with 474 patients aged 16–65 years old who met the inclusion criteria of schizophrenia-spectrum or bipolar disorder and classified the patients into two groups: a violent group with 191 patients and a nonviolent group with 283 patients. Violence was defined as the aggressive behavior of a patient with the necessity of special observation. We obtained all the required information retrospectively through patient files, applied the OxMIV tool on each subject, and compared the results of both groups. Sensitivity, specificity, and positive/negative predictive values were determined. We used logistic regression including variable selection and internal validation to identify relevant predictors of aggressive behavior in our cohort.

**Results:** The OxMIV score was significantly higher in the violent group [median 4.21%; Interquartile range (IQR) 8.51%] compared to the nonviolent group (median 1.77%; IQR 2.01%; p < 0.0001). For the risk of violent behavior, using the 5% cutoff for “increased risk,” the sensitivity was 44%, and the specificity was 89%, with a positive predictive value of 72% and a negative predictive value of 70%. Applying logistic regression, four items were statistically significant in predicting violent behavior: previous violent crime (adjusted odds ratio 5.29 [95% CI 3.10–9.05], p < 0.0001), previous drug abuse (1.80 [1.08–3.02], p = 0.025), and previous alcohol abuse (1.89 [1.21–2.95], p = 0.005). The item recent antidepressant treatment (0.28 [0.17–0.47]. p < 0.0001) had a statistically significant risk reduction effect.

**Conclusions:** In our opinion, the risk assessment tool OxMIV succeeded in predicting violent behavior in imprisoned psychiatric patients. As a result, it may be applicable for identification of patients with special needs in a prison environment and, thus, improving prison safety.

## Introduction

Violent behavior in individuals with severe mental disorders has been widely reported. Several studies and reviews from the United States (1, 2) and Europe (3–5) can verify this, and especially, two groups of patients (schizophrenia and bipolar disorder) are at higher risk of committing a violent crime compared to the general population (6, 7). This opinion is not agreed upon by all experts in the field, due to the vast majority of individuals diagnosed with schizophrenia never committing any act of violence (8).

Analyzing data of more than 24,000 cases of schizophrenia and related disorders, Fazel et al. pointed out that the adjusted odds ratio of adverse outcomes, including violent behavior, was 7.5 in men and 11.1 in women compared to the general population. They concluded that schizophrenia and related disorders are associated with increased rates of violent crime (5). In patients with bipolar disorders, the odds ratio for violent crime was 5 (6). Recent surveys have determined a variety of risk factors for aggressive behavior and violent crime in patients with schizophrenia and bipolar disorders, such as substance use disorder (SUD) (4, 7), young age (9), previous violent crime (10), male gender, and disadvantaged neighborhoods (11). Results of population-based studies suggest that there is an increased risk of violent offending and violent ideation in individuals with severe mental disorders and indicate a higher risk of homicide and violent crime, especially in individuals with schizophrenia (3). On the other hand, there are protective factors regarding violent behavior, including intelligence (12), self-control (13), intimate relationship (14), and social network (15). Modern risk assessment instruments such as the SAPROF (*Structured Assessment of Protective Factors for Violence Risk*) and the START (*Short-Term Assessment of Risk and Treatability*) are designed to consider the positive qualities of inmates and focus on resilient factors (16–18). Comparing patients with schizophrenia who showed violent behavior to individuals with the same diagnosis who were not violent, Ekinci and Ekinci reported that depressive symptoms were predictors for nonviolent behavior (19).

Regarding the results of epidemiological research on the increased incidence of violent behavior in patients with mental disorders, understanding the individual and situational risk factors for aggressive behavior seems to be crucial for the improvement of general safety and for the prevention of further stigmatization (9). To reduce and manage the risk of future violent behavior, one of the main approaches is the use of risk assessment tools. Over 200 different tools are currently available (20), with a wide range in applicability. Studies from the United Kingdom have evaluated that over 60% of general psychiatric patients and 80% of forensic psychiatric patients are routinely assessed for violent risk (21, 22). Despite the broad clinical application, there are just a few risk assessment instruments that have been externally validated (23).

In 2018, Ramesh et al. conducted a systematic review and meta-analysis on the use of risk assessment tools for predicting violent behavior in a forensic psychiatric hospital. Out of nine violence risk assessment instruments, only two (the *Bröset Violence Checklist* and the *Dynamic Appraisal of Dynamic Aggression*) demonstrated high accuracy for the prediction of violence (24). The most common tool, the HCR-20 (*Historical, Clinical, Risk Management-20*), had a moderate accuracy, while the PCL-R (*Psychopathy Checklist-Revised*) and the VRAG (*Violence Risk Appraisal Guide*) scored poorly on accuracy regarding the prediction of inpatient violent behavior (24). Further investigations into this area of research point to similar, debatable results: risk assessment tools have a range of accuracy (25) and a “large variation of the item content” (26) and are not designed for specific populations (24). As a result, the duration of stay in forensic institutions, often depending on the specific “risk” posed by the individual, may be longer than necessary (27), with social (28) and economic (29) consequences (25).

In 2017, based on data from 75,158 individuals, Fazel et al. developed a simple, web-based risk calculator (Oxford Mental Illness and Violence tool, OxMIV) to identify the future risk of violent behavior in patients with schizophrenia-spectrum and bipolar disorders. They developed a 16-item model for patient stratification into “low-risk” or “increased-risk” categories to identify the risk of violent offending within 12 months. With a sensitivity of 62% and a specificity of 94% in external validation, these results are the best in this field so far (10).

Although the total numbers of psychiatric beds has been declining in Europe since 1990, the number of institutionalized forensic psychiatric patients is increasing in Germany (30) and Europe (31). In addition, in six South American countries, the prison population is increasing, while the number of psychiatric beds has been decreasing since 1990 (32). These findings from Mundt et al. were consistent with the assumption of an association between the numbers of psychiatric beds and the sizes of prison populations as hypothesized by Sharples Penrose in 1939 (32). Regarding this development, there could be a connection to the increased tendency for violent behavior in patients with severe mental disorders compared to the general population (5).

## The Aim of the Study

The prediction of violent behavior through risk assessment tools is increasingly important for the treatment of mentally disordered patients and for the prevention of future offenses in general psychiatric and especially in forensic psychiatric hospitals. With this in mind, the aim of our study was to test the predictive validity of the risk assessment tool OxMIV for the first time in patients with schizophrenia-spectrum or bipolar disorders in the department of psychiatry at the Berlin prison hospital in Germany to improve prison safety by identifying patients with special needs in a prison environment.

For this study, we have considered two hypotheses. First, we assumed that violent patients have a significantly higher score in the prediction tool compared to nonviolent patients (7, 33, 34). Second, we hypothesized that a previous violent crime and a SUD have a statistically significant effect regarding the violent behavior of psychiatric patients (10, 35, 36).

## Materials and Methods

### Study Settings

For this retrospective study, a sample of 841 treatment episodes in the Berlin prison hospital between 1982 and 2017 was identified. Each treatment episode was defined as an inpatient stay by a patient in the department of psychiatry of the prison hospital in Berlin. For all identified treatment episodes, the patients were diagnosed with a schizophrenia-spectrum or a bipolar disorder (including schizophrenia, schizotypal, delusional, and other psychotic disorders) as well as previous comorbid depression in schizophrenia-spectrum disorder. The cases were pseudonymized with a personal number to protect private data. There were no connections between the names and the personal numbers.

**Figure 1:**
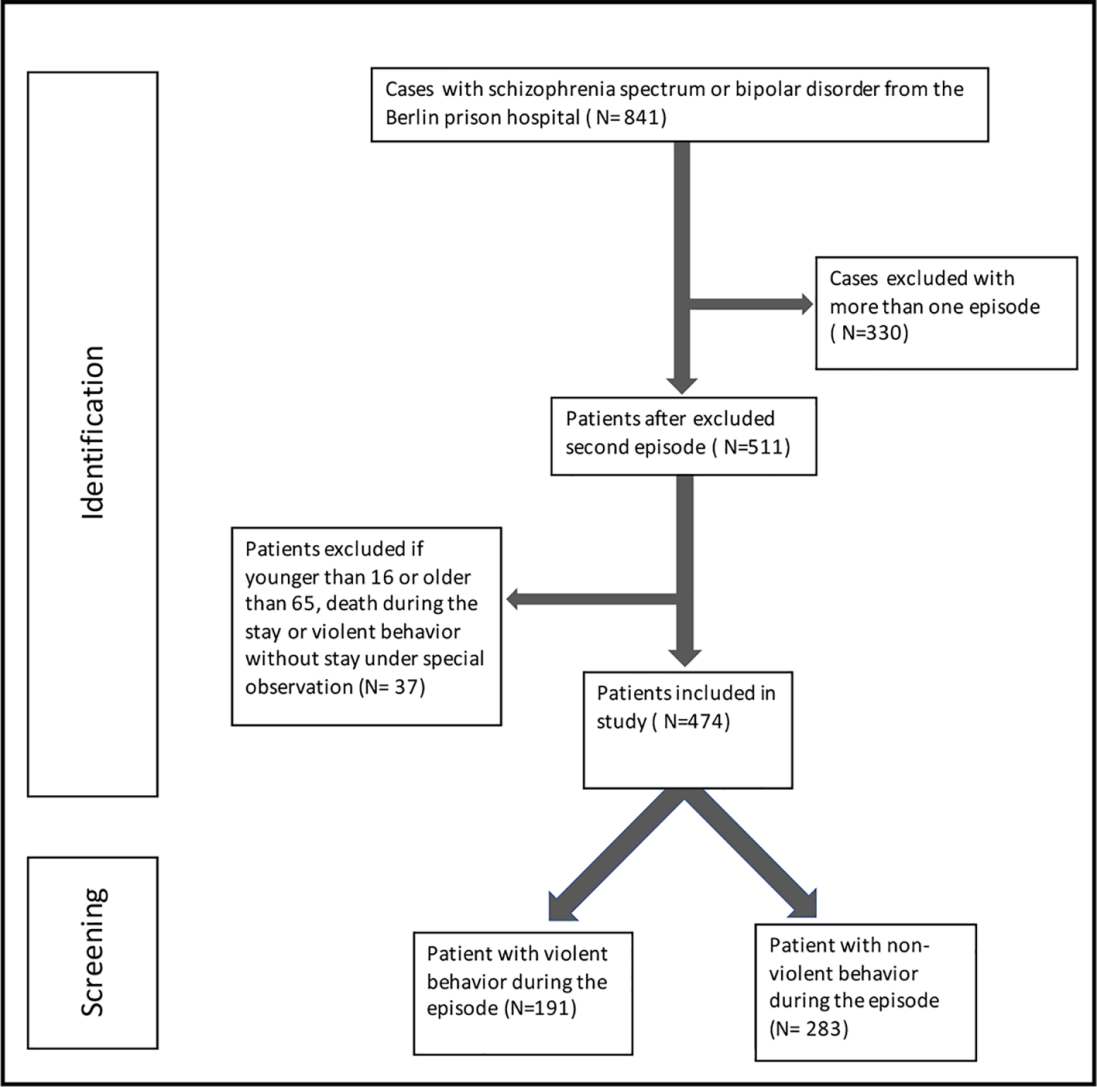
Systematic identification for patients of the Berlin prison hospital from 1982 till 2017.

In total, 841 treatment episodes where the patient had a schizophrenia-spectrum or bipolar disorder diagnosed could be assigned to 511 unique patients (see **Figure 1**). For patients with more than one inpatient stay, only the first stay in the prison hospital was included. Of these 511 patients, 36 patients had to be excluded from the study due to age (younger than 16 or older than 65) or death during the treatment. To ensure comparability within the violent group, one patient had to be excluded, because there was no necessity for special observation. Thus, 474 patients with a treatment episode at the Berlin prison hospital were included in our study (see **Figure 1**). In the last step, we formed two groups, a nonviolent group and a violent group; 191 patients who, for a certain period of time, had to stay under special observation due to violent behavior were included in the violent group, and 283 patients who demonstrated no violent behavior during their stay were assigned to the nonviolent group (see **Figure 1**).

### Procedures

The OxMIV is a prediction tool to identify those patients who are at low risk of violent offending. On the basis of 75,158 individuals with a schizophrenia-spectrum or bipolar disorder, the tool consists of 16 items, such as previous violent crime, drug and alcohol abuse, and socioeconomic and sociodemographic information (10). The final score classifies individuals into either a low-risk (<5%) or an increased-risk (>5%) group with a set point of 20%.

The required information on the 16 items was obtained by looking through the patient files for the whole treatment period. It is important to point out that we received the necessary information on the items previous drug abuse, previous alcohol abuse, previous self-harm, education level, parental violent crime, parental drug or alcohol use, sibling violent crime, recent treatment, personal income, and recent benefits through the admission interview with the medical doctor in the prison ward. These admission interviews take place within the first few days of imprisonment. For the item previous violent crime, we used official information from the Federal Central Criminal Register (Bundeszentralregister), which includes past convictions and is also available within the first days of imprisonment. In terms of the items parental violent crime, parental drug or alcohol use, and sibling violent crime, not all information was available for every patient. For these cases, we had to select “unknown.” We defined schizophrenia-spectrum disorder, bipolar disorder, and comorbid depression using the International Classification of Diseases (ICD), Ninth Version (ICD-9) (295, 297–299 excl. 299A, 296 excl. 296.2; 296 excl. 296D, 296.2, 300.4; 296D, 300E, 311 1979–1998) or Tenth Version (ICD-10) (F 20–29, F 30–31; F32–F34.1 1998–2018). In addition, comorbid alcohol and drug abuse disorders were based on the ICD-9 or Tenth Version (ICD-10) (F 11–19; 1998–2018).

All of the patients were screened *via* OxMIV. If the score was above the set point of 20%, we calculated the result with the formula by Fazel et al. (9) using Excel version 14.7.7. If one or more variables were unknown, OxMIV calculated a range of risk levels. To generate a score, the highest and lowest risk levels were calculated to determine the mean.

### Outcomes and Definition

The primary outcome was the score of the risk assessment tool OxMIV. We defined violent behavior as any physical violence (kicking, biting, hitting, scratching) or verbal violence (psychological, emotional, threatening, insulting) against another person or a group, as well as the inventory (destruction, arson of detention rooms) that led to the necessity for special observation of the patient. This definition is based on the definition of violence from the World Health Organization (WHO) (37) and considerations from Wolff et al. (38). The WHO defines violence as “the intentional use of physical force or power, threatened or actual, against oneself, another person, or against a group or community, that either results in, or has a high likelihood of resulting in injury, death, psychological harm, maldevelopment, or deprivation” (37).

### Statistical Analysis

Continuous parameters are shown as mean (SD) and categorical parameters as absolute frequencies and percentages. Continuous variables were compared between the groups using the t-test. Before that, the normality assumption was tested with the Shapiro–Wilk test. Variance homogeneity was tested with the Levene test. In case of violated assumptions, the Mann–Whitney U test was used. Categorical parameters were compared using the chi-square test. In 16 (6%) patient cases with an OxMIV score above the cutoff limit of 20%, we had to calculate each score using the given formula. This concerned 13 cases in the violent group and three cases in the nonviolent group. Using the 5% cutoff, we calculated the sensitivity, specificity, as well as the positive and negative predictive value. Further, the area under the receiver operating characteristic (ROC) curve was determined.

Due to the different setting and outcome in the original OxMIV study, we investigated a logistic regression model including 14 of the 16 items from the OxMIV. Two variables were excluded since all participants were male and currently in inpatient treatment. The items parental violent crime, parental drug or alcohol use, and sibling violent crime had missing values, and thus, we applied multiple imputation (m = 20 imputations), including all of the other parameters and the outcome in the imputation model. Further, we used variable selection in each imputed data set using backward selection with likelihood ratio tests to identify a sparse model for the prediction of violent behavior in our cohort. A variable remained in the model if it was selected in at least 80% of the imputations. The 14-item model and the selected model were internally validated using the Val-MI approach by Wahl et al. (39) with 20 imputations and 50 bootstrap samples.

A p value smaller than 0.05 was considered significant, although all results have to be interpreted as exploratory due to the nature of the study. All analyses and calculations were performed with the statistical program SPSS, version 25.0.0, and R, version 3.5.0 (40).

## Results

### Descriptive Characteristics

Of 474 patients with schizophrenia-spectrum or bipolar disorder, 283 (60%) were nonviolent, and 191 (40%) were violent with the need for special observation during their stay in the psychiatric ward (see **Table 1**). The mean age was 32 [standard deviation (SD) = 9]. Regarding the diagnosis, 438 (92%) patients had schizophrenia, 25 (5%) had a bipolar disorder, and 11 (3%) had a schizophrenia-spectrum disorder with a comorbid depression. Out of 474 patients, 104 (22%) committed a previous violent crime, and 313 patients (66%) had a drug abuse disorder, and 220 (46%) had an alcohol abuse disorder. These two disorders were the common comorbid diagnosis. Concerning the topic of medication, overall, 436 (92%) of 474 patients had a recent antipsychotic treatment, and 143 (30%) had a recent dependence treatment. Overall, 147 (31%) had a recent antidepressant treatment.

**Table 1:** Descriptive data from the nonviolent and violent groups in the Berlin prison hospital divided into risk factors.

	Nonviolent n = 283	Violent n = 191	Total n = 474	p value
Age	33 [10]	31 [9]	32 [8]	0.510
Male sex	283 (100%)	191 (100%)	474 (100%)	–
Previous violent crime	28 (10%)	76 (40%)	104 (22%)	<0.001
Previous drug abuse	167 (59%)	146 (76%)	313 (66%)	<0.001
Previous alcohol abuse	109 (39%)	111 (58%)	220 (46%)	<0.001
Previous self-harm	34 (12%)	21 (11%)	55 (12%)	0.734
Education level				0.888
• Secondary	240 (85%)	165 (86%)	405 (85%)	
• Upper secondary	26 (9%)	16 (8%)	42 (9%)	
• Post secondary	17 (6%)	10 (5%)	27 (6%)	
Parental drug or alcohol use	19 (7%)	9 (5%)	28 (6%)	0.600
Parental violent crime	6 (2%)	15 (8%)	21 (4%)	0.001
Sibling violent crime	1 (0.4%)	3 (2%)	4 (1%)	0.110
Recent treatment				
• Antipsychotic	259 (92%)	177 (93%)	436 (92%)	0.651
• Antidepressant	116 (41%)	31 (16%)	147 (31%)	<0.001
• Dependence	78 (28%)	65 (34%)	143 (30%)	0.132
Personal income				0.088
• First and second deciles	179 63%)	173 (91%)	352 (74%)	
• Third and fourth deciles	91 (32%)	70 (37%)	161 (34%)	
• Fifth to tenth deciles	13 (5%)	4 (2%)	17 (4%)	
Inpatient	283 (100%)	191 (100%)	474 (100%)	–
Benefit recipient	62 (22%)	54 (28%)	116 (24%)	0.114
Diagnosis				0.030
• Schizophrenia-spectrum disorder	262 (92%)	176 (92%)	438 (92%)	
• Bipolar disorder	11 (4%)	14 (7%)	25 (5%)	
• Comorbid depression	10 (4%)	1 (1%)	11 (2%)	

Data are shown as n (%) and mean [SD].

There were statistically significant differences between the two subgroups (violent/nonviolent) regarding the items diagnosis (p = 0.030), previous violent crime (p < 0.0001), drug abuse disorder (p < 0.0001), alcohol abuse disorder (p < 0.0001), parental violent crime (p = 0.001), and recent antidepressant treatment (p < 0.0001; see **Table 1**).

### Comparison Between Risk Levels in OxMIV Score

**Figure 2:**
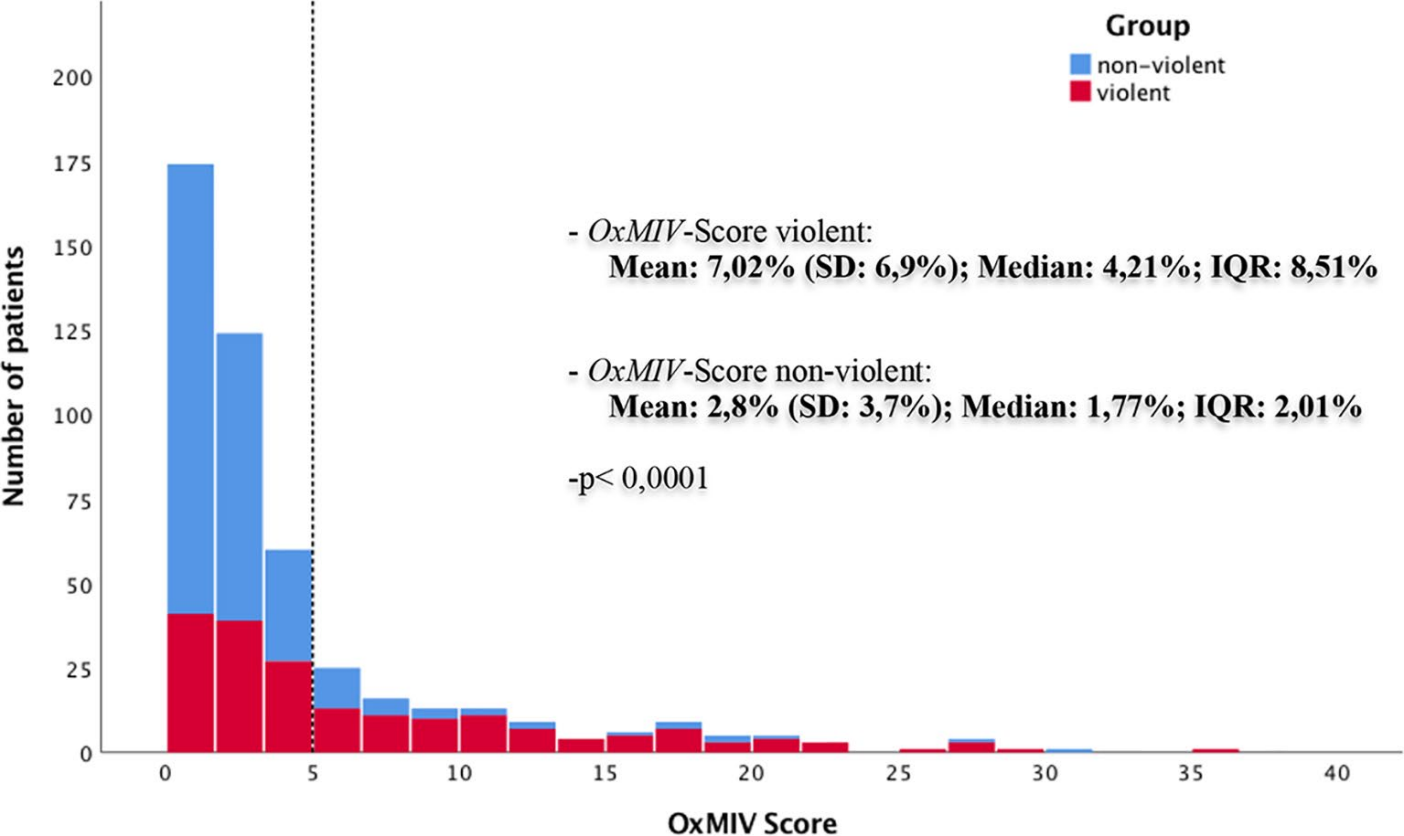
Distribution of the Oxford Mental Illness and Violence tool (OxMIV) score in the nonviolent and violent groups with the cutoff set at 5% for “increased risk” of violent behavior (dashed line).

**Figure 3:**
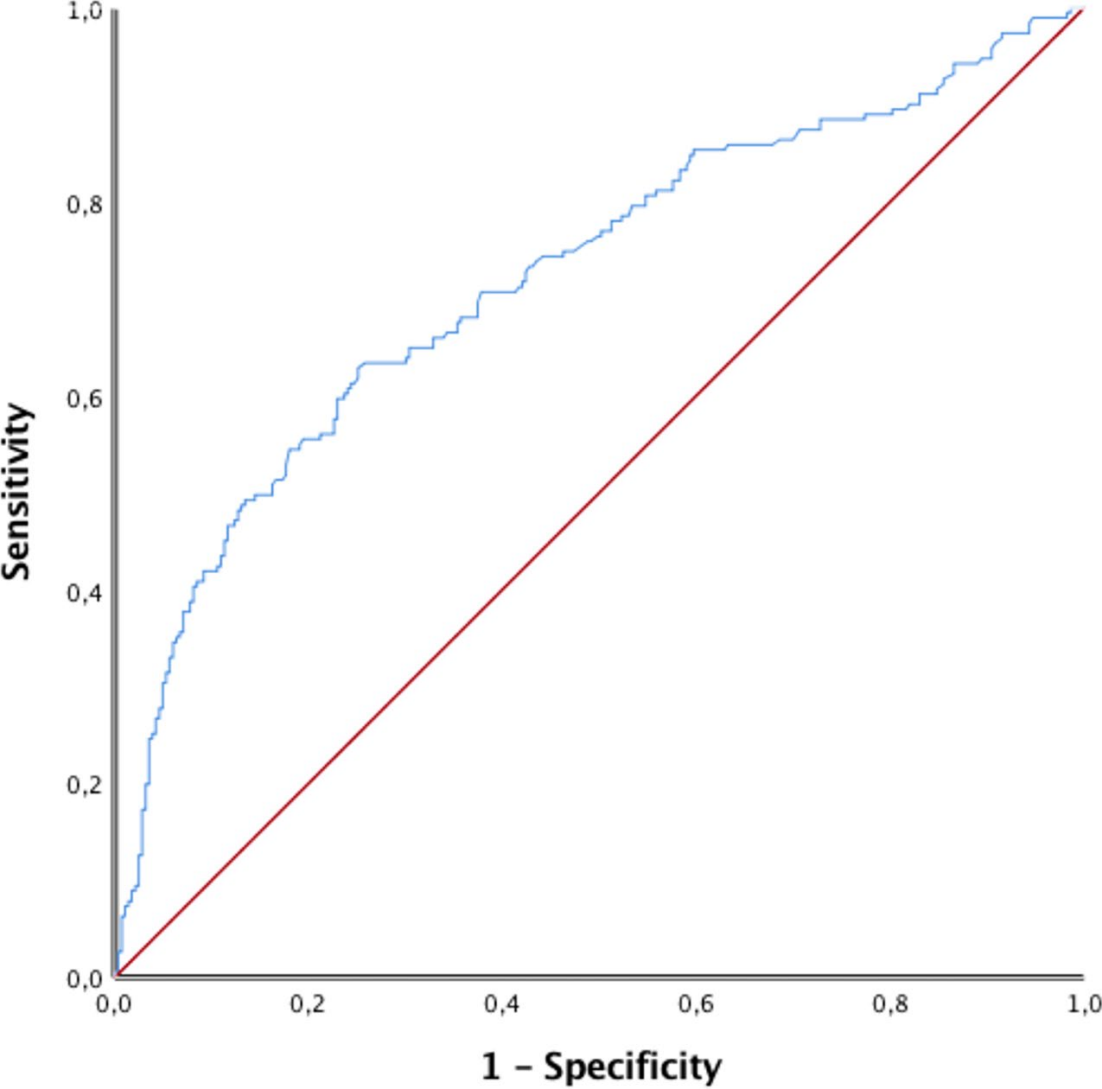
Receiver operating characteristic (ROC) curve for the prediction of violent behavior.

The results of the OxMIV score were significantly higher in the violent group compared to the nonviolent group (p < 0.001). The risk levels were divided into a low-risk and increased-risk group, with a defined cutoff at 5%. In the violent group, the median was 4.21%, and the IQR was 8.51%. In the nonviolent group, the median was 1.77%, and the IQR was 2.01%, as seen in **Figure 2**. Out of 474 patients, 358 (76%) patients were classified as low risk, and 116 (24%) patients were at increased risk. In the nonviolent group, 251 of 283 (89%) were categorized as low risk compared to 107 of 191 (56%) in the violent group. Regarding increased risk, 84 of 116 (72%) of the violent group compared to 32 of 116 (28%) in the nonviolent group had an OxMIV score >5% (p < 0.0001; see **Table 2**). For a 5% cutoff for an increased risk for violent behavior, the sensitivity was 44%, and the specificity was 89%, with a positive predictive value of 72% and a negative predictive value of 70%, as seen in **Table 2**. Through the receiver operating characteristic curve (ROC curve), we calculated an area under the curve (AUC) value of 0.72 (see **Figure 3**).

**Table 2:** Two-by-two table using the OxMIV score and the 5% cutoff to derive sensitivity, specificity, positive prediction value, and negative prediction value to identify “increased-risk” patients for violent behavior during their stay.

		Violent behavior during stay		
		Yes	No	Total
OxMIV score >5%	Yes	84	32	**116**
	No	107	251	**358**
	Total	**191**	**283**	474

OxMIV, Oxford Mental Illness and Violence tool.

### Binomial Logistic Regression

In the 14-item model, the strongest significant predictors of violent behavior during the stay were previous violent crime (adjusted odds ratio 5.29 [95% CI 3.10–9.05], p < 0.0001), previous drug abuse (1.80 [1.08–3.02], p = 0.025), and previous alcohol abuse (1.89 [1.21–2.95], p = 0.005). The strongest predictor against violent behavior was recent antidepressant treatment (0.28 [0.17–0.47], p < 0.0001; see **Table 3**). The optimism-corrected AUC (0.75), Brier score (0.20), and pseudo-R² (0.18) indicated fair performance, while the calibration intercept (0.35) and slope (0.82) showed poor performance. The Hosmer–Lemeshow test was nonsignificant in all 20 imputed data sets for the 14-item model.

**Table 3:** Association between risk factors and violent behavior from logistic regression after multiple imputation.

	Coefficient	Standard error	p value	Adjusted odds ratio	95% CI	
Age	−0.02	0.01	0.171	0.98	0.96	1.00
Previous violent crime	1.66	0.27	<0.0001	5.29	3.10	9.05
Previous drug abuse	0.59	0.26	0.025	1.80	1.08	3.02
Previous alcohol abuse	0.63	0.23	0.005	1.89	1.21	2.95
Previous self-harm	−0.53	0.37	0.151	0.59	0.28	1.21
Education level						
- Lower secondary	1 (reference)					
- Upper secondary	0.32	0.42	0.444	1.38	0.60	3.17
- Postsecondary	0.16	0.48	0.737	1.18	0.46	3.01
Parental drug or alcohol use	−0.29	0.45	0.513	0.75	0.31	1.80
Parental violent crime	0.71	0.40	0.076	2.04	0.93	4.50
Sibling violent crime	−0.01	1.01	0.990	0.99	0.13	7.34
Recent antipsychotic treatment	0.07	0.42	0.861	1.08	0.48	2.44
Recent antidepressant treatment	−1.26	0.26	<0.0001	0.28	0.17	0.47
Recent dependence treatment	0.05	0.24	0.831	1.05	0.66	1.69
Benefit recipient	0.31	0.26	0.233	1.37	0.82	2.29
Personal income	−0.18	0.14	0.203	0.84	0.63	1.10
Constant	−0.36	0.74	0.628	−	−	−

After variable selection, previous violent crime (adjusted odds ratio 5.08 [95% CI 3.02–8.55], p < 0.0001), previous drug abuse (2.09 [1.30–3.37], p = 0.002), previous alcohol abuse (1.76 [1.15–2.70], p = 0.009), parental violent crime (2.04 [1.00–4.19], p = 0.051), and recent antidepressant treatment (0.28 [95% CI 0.17–0.46], p < 0.0001) remained in the model (see **Table 4**). The adjusted odds were almost unchanged, indicating a stable predictive ability independent of the other considered variables in the 14-item model. The optimism-corrected AUC (0.76), Brier score (0.19), and pseudo-R² (0.20) were slightly improved, still indicating fair performance, while calibration (intercept = 0.06, slope = 0.96) was greatly improved, showing good calibration of the model. The Hosmer–Lemeshow test was nonsignificant in all 20 imputed data sets for the selected model. The pseudo-R² was 0.203.

**Table 4:** Association between risk factors and violent behavior from logistic regression after multiple imputation and variable selection.

	Coefficient	Standard error	p value	Adjusted odds ratio	95% CI	
Previous violent crime	1.63	0.27	<0.0001	5.08	3.02	8.55
Previous drug abuse	0.74	0.24	0.002	2.09	1.30	3.37
Previous alcohol abuse	0.57	0.22	0.009	1.76	1.15	2.70
Parental violent crime	0.71	0.36	0.051	2.04	1.00	4.19
Recent antidepressant treatment	−1.27	0.25	<0.0001	0.28	0.17	0.46
Constant	−1.31	0.24	<0.0001	0.27	0.17	0.43

## Discussion

The purpose of this study was to test the predictive validity of the risk assessment instrument OxMIV in patients with a schizophrenia-spectrum or bipolar disorder in a German prison hospital. We analyzed 474 patient files from the data bank of the Berlin prison hospital and divided these into two groups: a violent and a nonviolent group. We (retrospectively) performed a risk assessment in all 474 patients using the prediction tool OxMIV, which is a 16-item model including criminal and personal background information, as well as sociodemographic and clinical risk factors. Out of 474 patients, 191 demonstrated violent behavior during their stay in comparison to 283 nonviolent patients. As hypothesized, patients who had demonstrated violent behavior had a significantly higher OxMIV score compared to the nonviolent group, with a fair performance in internal validation.

As assumed, the items previous violent crime, previous drug abuse, and previous alcohol abuse had a significant impact on the occurrence of violent behavior during inpatient treatment. This is in line with previous results in this field (3–5, 9, 10, 33, 41) and highlights further the strong interactions between severe mental disorders and substance abuse. In our results, the item recent antidepressant treatment had a risk reduction effect on violent behavior. This is in line with the findings of Fazel et al., who described a positive effect of antipsychotic medication and mood stabilizers on violent behavior and the occurrence of violent crime in over 82,000 patients with mental disorders (42, 43).

Using a logistical regression model after variable selection led to the identification of five items that demonstrated a significant effect on the prediction of violent behavior in our cohort. Thus, it may be discussed whether the risk assessment tool could be reduced to a five-item model consisting of: previous violent crime, previous drug abuse, previous alcohol abuse, parental violent crime, and recent antidepressant treatment. In light of the good performance in internal validation of the reduced five-item model, compared to the fair performance of the 16-item model, these findings lead to the assumption that the risk assessment tool OxMIV could be adjusted for male patients in a forensic psychiatric prison ward. However, to clarify these questions, further studies are needed.

There are certain differences when comparing our results to the results of the OxMIV study by Fazel et al. (10). They found three strong predictors of violent behavior/offending that (in part) differed from our results: previous violent crime, male gender, and age (10). Clearly, it was not possible for us to consider the item “gender” because of our study population, which consisted solely of male patients. Regarding the age variable, two studies on risk factors for prison violence found the item “older age” to be a protective factor (9, 10). In terms of the item “age,” we did not find this to be a significant protective factor for violent behavior in our study. In our opinion, the results of this study might have several implications for the treatment of patients in the forensic psychiatric ward. Due to the uncomplicated use of this risk assessment tool, not only medical doctors are able to categorize patients into low-risk and increased-risk groups, but also other clinical staff, including psychologists and trained nursing staff. In line with our findings, for the specific prison setting, it may be sufficient to focus on the five items that demonstrated a significant effect on predicting violence (see above), thus increasing practicability even further. It is known that mental health professionals are at a greater risk to be victims of violent offenses (44, 45). Analyzing a 12-month period of time, Foster et al. indicated a 1-in-10 chance per year to be attacked in a psychiatric hospital in the United Kingdom (46). Therefore, it is indispensable to have specific tools and skills to handle and prevent violent behavior (47). However, it is equally important to mention that this risk assessment tool is primarily intended as an adjunct for clinical decision-making and not as an isolated diagnostic device.

To our knowledge, the positive prediction value of 72% is the highest value in risk assessments so far. A 2012 systematic review on the nine most commonly used risk assessment tools in forensic wards reported a median positive prediction value of 41% (IQR: 27–60%) (25). As a consequence, nearly two-thirds of the patients in our cohort who demonstrated violent behavior by passing the 5% cutoff score were screened as increased risk by the OxMIV. Regarding the results from the original OxMIV study by Fazel et al., the positive prediction score was 11%, and the negative prediction score was 99% (10). Thus, nearly all of the patients from the general psychiatric service who had a score under the 5% cutoff did not commit a violence offense in the following 12 months. As a reason, the clinical implication of the original OxMIV was to identify low violence risk (10). Due to the specific nature of our patients (all male, mean age of 32), with a higher baseline risk (overall, 22% previous violent crime, 66% drug abuse, 46% alcohol abuse), we think that these aspects had a major influence on the results regarding the positive predictive value. Although we had a very specific cohort, our results suggested that the OxMIV may be used to identify violent behavior in high-risk patients of forensic psychiatric wards as well. As a consequence, the security in forensic wards for fellow inpatients and especially for staff members may be generally increased, while in addition, more specific treatment options regarding possibly aggressive patients could be implemented (e.g. group size, staffing ratio).

The reduction of future violent offenses by psychiatric patients after discharge may be possible through early identification of risk scenarios and specific preventive measures such as psychotherapeutic and psychopharmacological interventions. While static items of the OxMIV (parental violent crime, previous violent crime, gender, age) are not adjustable, our results suggest that targeting the changeable items of this tool by working on current and future treatment options with these patients (e.g. antidepressants, CBT, motivational interviewing for SUD) may have a positive influence on the prevention of violent behavior. Due to the known risk of violent offenses after discharging patients with severe mental disorders (6, 10), the prediction and especially the treatment of violent behavior in prison and in forensic wards is beneficial not only for the patients but also for society as a whole (47, 48).

A major limitation of this study is that only male patients were included due to our specific setting. Despite its free and easy use, there are also limitations of the prediction score OxMIV. On the one hand, it should be discussed that using the OxMIV tool for active risk management in a clinical prison hospital setting may be insufficient. The 16 items are all of a retrospective nature and not specifically designed for mapping an individual treatment process. Furthermore, the items age, previous violent crime, and male sex had a disproportionate effect on the total score. A patient (always) showed an increased risk (5.3–9.9%) if he was between 20 and 35 years old and male and had a history of previous violent crimes. The other prediction variables, in comparison, did not have the same power. On the other hand, as mentioned above, the items on the recent treatment of the patient do point to possibilities regarding risk management without the option to actively track the change in risk while treating the patient, due to missing specific clinical items. Another limitation of our study was the retrospective design, which may have led to various biases, such as recall bias or the present state effect (49). A further limitation is the fact that we did not check for interrater reliability as well as multicollinearity between the items. In terms of multicollinearity, we accepted the disturbance between the items as well as a wider range in the confidence interval and added a variable selection process to reduce the number of parameters for our setting. Still, multicollinearity might be a problem, but in order to yield a predictive model, we consider this problem as minor. Another limitation is that we conducted our study in just one psychiatric department in one prison hospital in one country. Despite the fact that inpatient forensic psychiatric health care differs thoroughly in high-income countries such as Germany, Great Britain, and the Netherlands, further research is necessary (48).

In summary, to the best of our knowledge, for the first time in Germany, we tested the predictive validity of the risk assessment tool OxMIV in patients with schizophrenia-spectrum or bipolar disorders in a prison hospital. Even if there are different kinds of studies in Germany that are dealing with specific overviews of mental disorders in the criminal justice system (50), specific treatment in forensic psychiatric wards (51), and suicide rates (52), there is less research on violent behavior and its assessment in patients in prison settings. Despite the significant results of this study, further studies in different countries are needed.

## Ethics Statement 

According to current legal regulation, the study was approved by the local ethics committee at Charité–Universitätsmedizin Berlin.

## Author Contributions

VN and AO designed the study. VN, PS, and AO collected the data. VN, AV, RR, and AO analyzed and interpreted the data. VN, AV, and AO wrote the initial draft of the manuscript. VN, PS, and AO had full access to all the data in the study and take responsibility for the integrity of the data and the accuracy of data analysis. All authors have contributed to, read, and approved the final version of the manuscript.

## Funding

The authors declare that, except for income received from their primary employer, no financial support or compensation has been received from any individual or corporate entity over the past 12 months for research or professional service related to this study and there are no personal financial holdings that could be perceived as constituting a potential conflict of interest.

## Conflict of Interest Statement

The authors declare that the research was conducted in the absence of any commercial or financial relationships that could be construed as a potential conflict of interest.
